# High-frequency ultrasound in clinical dermatology: a review

**DOI:** 10.1186/s13089-021-00222-w

**Published:** 2021-04-20

**Authors:** Jack Levy, Devon L. Barrett, Nile Harris, Jiwoong Jason Jeong, Xiaofeng Yang, Suephy C. Chen

**Affiliations:** 1 Department of Dermatology, Emory University School of Medicine, Atlanta, GA USA; 2 Department of Radiation Oncology, Emory University School of Medicine, Atlanta, GA USA; 3 Department of Biomedical Informatics, Emory University, Atlanta, GA USA; 4 Winship Cancer Institute, Emory University, Atlanta, GA USA; 5 Department of Dermatology, Duke University School of Medicine, Duke Clinic, 40 Duke Medicine Cir, Clinic 3K, Durham, NC 27710-4000 USA

**Keywords:** High-frequency ultrasound, Dermatology, Diagnostic imaging, Ultrasonography

## Abstract

**Background:**

Ultrasound was first introduced in clinical dermatology in 1979. Since that time, ultrasound technology has continued to develop along with its popularity and utility.

**Main text summary:**

Today, high-frequency ultrasound (HFUS), or ultrasound using a frequency of at least 10 megahertz (MHz), allows for high-resolution imaging of the skin from the stratum corneum to the deep fascia. This non-invasive and easy-to-interpret tool allows physicians to assess skin findings in real-time, enabling enhanced diagnostic, management, and surgical capabilities. In this review, we discuss how HFUS fits into the landscape of skin imaging. We provide a brief history of its introduction to dermatology, explain key principles of ultrasonography, and review its use in characterizing normal skin, common neoplasms of the skin, dermatologic diseases and cosmetic dermatology.

**Conclusion:**

As frequency advancements in ultrasonography continue, the broad applications of this imaging modality will continue to grow. HFUS is a fast, safe and readily available tool that can aid in diagnosing, monitoring and treating dermatologic conditions by providing more objective assessment measures.

## Introduction

Dermatology is among the rare specialties of medicine in which the organ of interest can be readily examined and biopsied. As a consequence, histology has become the gold standard for many dermatologic diagnoses. It has also allowed for clinicopathologic correlation to guide therapeutics and advance prognostics [1]. In the last few decades, a number of imaging modalities have been introduced to augment clinical exam or obviate the need for histology [2]. Some examples include total body digital photography (TBDP), dermoscopy, reflectance confocal microscopy (RCM), optical coherence tomography (OCT), and ultrasound. In this review, we discuss how ultrasound fits into the landscape of skin imaging. We provide a brief history of its introduction to dermatology, explain key principles of ultrasonography, and review its use in characterizing normal skin, common neoplasms of the skin, dermatologic diseases and cosmetic dermatology.

## History of ultrasound in medicine

Sonography dates back to the late 1700s when two Italian zoologists discovered the acoustic orientation sense of bats [3]. Nearly a century later, the Curie brothers presented the piezoelectric effect of sound waves on natural quartz [4]. It was their observation that a force applied to the crystal produced an electric discharge. Conversely, an alternating electric current applied to a crystal produces vibrations that generate high-frequency sound waves called ultrasound [4, 5]. Over a half-century later, the technique was adapted by both the American military as SONAR and by an American Neurologist, Karl Dussik, who used ultrasound to detect brain tumors in patients [5].

Ultrasound emerged in the practice of dermatology around 1980 when two independent groups used the technique to measure skin thickness in normal and diseased skin [6]. Since then, the practice has gained popularity in several European and South American countries that incorporate its use in resident training and where its application is reimbursable [7, 8]. As the technology has improved, so has its utility. In an early study of over 4300 skin lesions, Wortsman et al. demonstrated the ability of higher frequency ultrasound (7–15 MHz) to significantly improve diagnostic accuracy. Compared to clinical examination alone, ultrasound corrected the referring diagnosis in 17% of cases and detected 3 missed malignancies [9]. The application of ultrasound in dermatology is continuously evolving and increasingly studied [10, 11].

## Ultrasound technology and how it works

Ultrasound allows for high-resolution imaging of the skin from the stratum corneum down to the deep fascia [12]. Newer ultrasound technologies, including high-frequency ultrasound (HFUS, between 20 and 30 MHz) and ultra high-frequency ultrasound (UHFUS, > 30 MHz), have further enhanced imaging clarity and expanded upon the myriad applications for use of ultrasound in dermatology [11, 13, 14]. With fast acquisition times, ultrasound images are produced in real-time, allowing for physical adjustments and optimal imaging. Many devices include color Doppler analysis for characterization of blood flow and vessel morphology. It is readily available and can be easily incorporated into clinical practice [10, 11, 15].

The main component of the ultrasound device is the transducer—typically a handheld device containing thousands of piezoelectric crystals. These crystals, when stimulated by an electric voltage, generate millions of acoustic waves that pass through tissue and are reflected back to the transducer. As the waves return to the transducer, they are converted back into electrical energy interpreted by a computer. The computer then generates a one-dimensional line graph in amplitude mode (A-mode) that can be used to interpret echogenicity at various distances from the probe. In brightness mode (B-mode) an image is generated with different intensities of brightness.

These varying intensities are dependent upon the ultrasound waves’ ability to pass through the tissue. Bright or hyperechoic regions represent an abrupt increase in tissue density while dark or hypoechoic areas represent a decrease or no change in tissue density. A black anechoic region represents an area of tissue from which virtually no waves are reflected [5]. Although dense structures reflect a greater number of waves, resulting in a hyperechoic ultrasonographic structures, they may also generate an artifactual acoustic shadow due to attenuation of the ultrasound waves at the interface between two tissues of varying densities [13]. Another common artifact, acoustic enhancement, can appear as a result of relatively unimpeded passage of ultrasound waves through a preceding area of low acoustic impedance. This results in the appearance of a brighter structure deep to the area of low impedance [16, 17].

The resolution and penetration of an ultrasound image depends on the frequency of the acoustic waves produced. Traditional lower-frequency ultrasound devices are ideal for evaluation of internal organs like the liver and lungs due to their penetration ability. HFUS and UHFUS waves, on the other hand, are unable to travel easily through tissue, but provide higher-resolution, superficial detail. This makes HFUS and UHFUS ideal for visualizing a superficial organ like the skin [5, 10, 11, 15, 18, 19]. Given the diverse range of probe frequencies and the variation of dermal thickness in different regions of the body, specific frequencies should be considered for optimal visualization of lesions in various anatomic locations [15].

### Normal skin

**Fig. 1:**
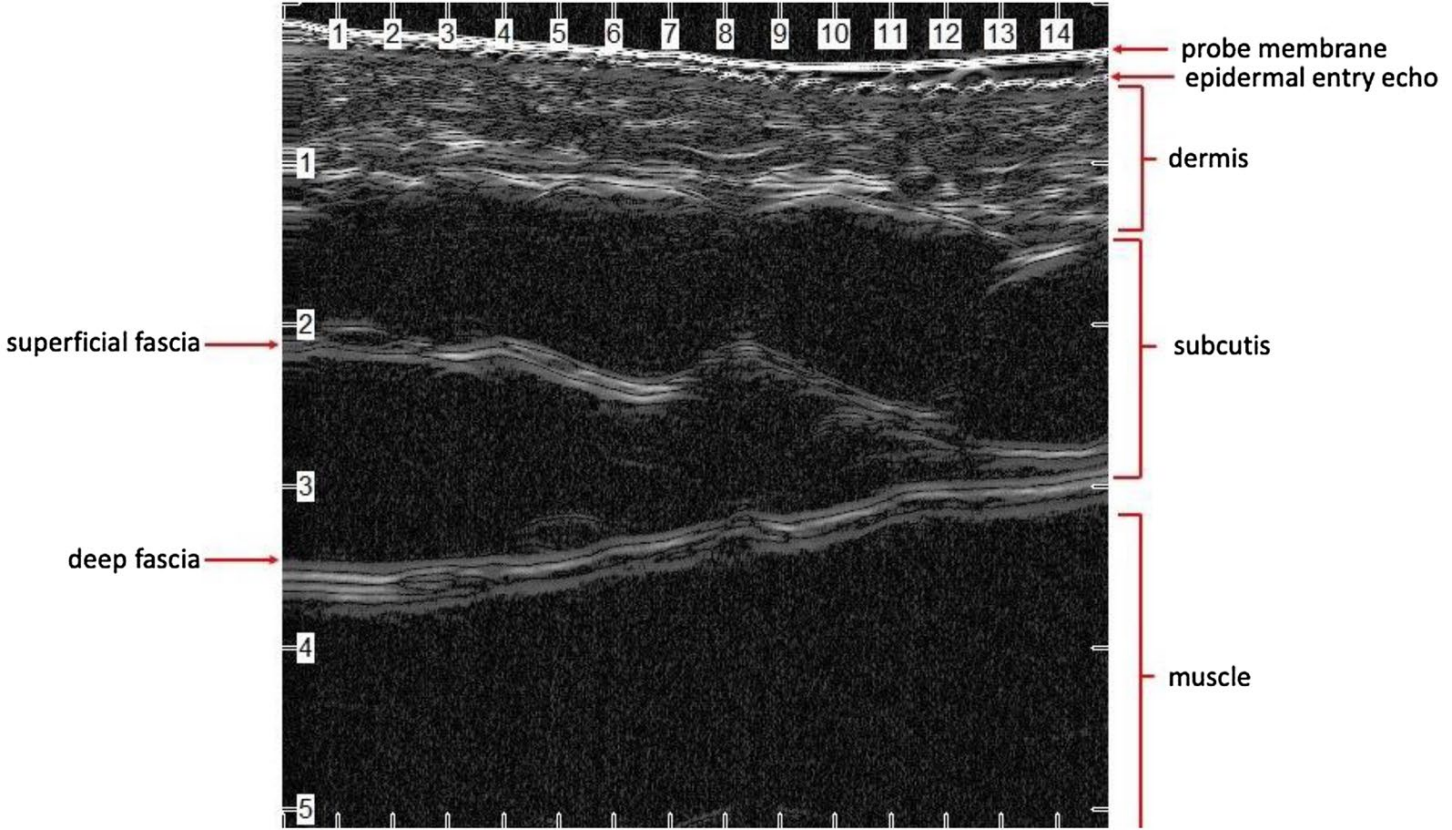
Normal skin from the anterior arm using a 50-MHz HFUS probe (Longport, Inc. EPISCAN). Note the fine hyperechoic epidermal entry echo. Deep to the entry echo is the hyperechoic dermis measuring about 1 millimeter (mm) with thicker horizontal bands in the deep dermis corresponding to the organized, horizontally arrayed collagen bundles in the reticular dermis. Deep to the dermis is the subcutis composed primarily of hypoechoic fat globules. Within this layer is the hyperechoic superficial fascia. Deep to the subcutis is the hyperechoic deep fascia overlying the biceps muscle

One of the first dermatologic applications of ultrasound measured the thickness of healthy skin [20]. While this original study used a 15-MHz transducer (and not meeting current standards for HFUS), the study was among the first to highlight the benefit of higher frequencies. Subsequent research has demonstrated that the three layers of the skin have distinct echogenicity, or corresponding levels of brightness (Fig. 1) [21]. The epidermis is mainly composed of keratin, a dense, fibrous structural protein that strongly reflects ultrasound waves; therefore, the epidermis is hyperechoic. The dermis is also hyperechoic, though to a lesser extent than the epidermis, due to its high content of collagen. Finally, the subcutis is hypoechoic due to the presence of fat lobules that allow for the unimpeded passage of ultrasound waves [2, 21].

The dermis can be further distinguished on ultrasound based on the arrangement of collagen and elastin bundles; the upper 20% of the hyperechoic dermis corresponds to the papillary dermis and contains irregularly arranged, thinner collagen and elastin bundles. The lower 80% of the dermis is made up of more regularly arranged collagen and elastin. Correspondingly, ultrasound at frequencies > 10 MHz demonstrates a reticular dermis with hyperechoic strands of thicker collagen bundles interposed with hypoechoic extracellular matrix [2, 5, 22].

An important consideration when using ultrasound to study healthy skin is persistence of a well-defined high-amplitude echo between the ultrasound gel and the corneal layer of the epidermis. This has been attributed to the impedance jump of the ultrasound waves from the ultrasound gel to the callused epidermal cells [2]. This reflection is thus referred to as the epidermal entry echo. Thus, the epidermal entry echo is not a direct visualization of the epidermis per se, but an artifact representing the epidermis [23].

Ultrasound studies examining healthy skin have demonstrated that while skin thickness is inversely proportional to age, echogenicity tends to increase with age corresponding to increase collagen production [24–28]. One notable exception is sun-damaged skin, as first described in 1988 by Querleux et al., where there is presence of a subepidermal low-echogenic band (SLEB). The SLEB was described as a dark band subjacent to the epidermal entry echo [29]. Though not specific to sun damage, its presence in more than 50% of adults over the age of 40 and its prominence in highly sun-exposed areas like the face and dorsal forearms have been correlated to solar elastosis, a histologic marker of cutaneous sun damage [25]. Similar findings have been recently demonstrated in patients treated with radiotherapy for head and neck cancers [30].

#### Glabrous skin, hair follicles, and the scalp

**Fig. 2:**
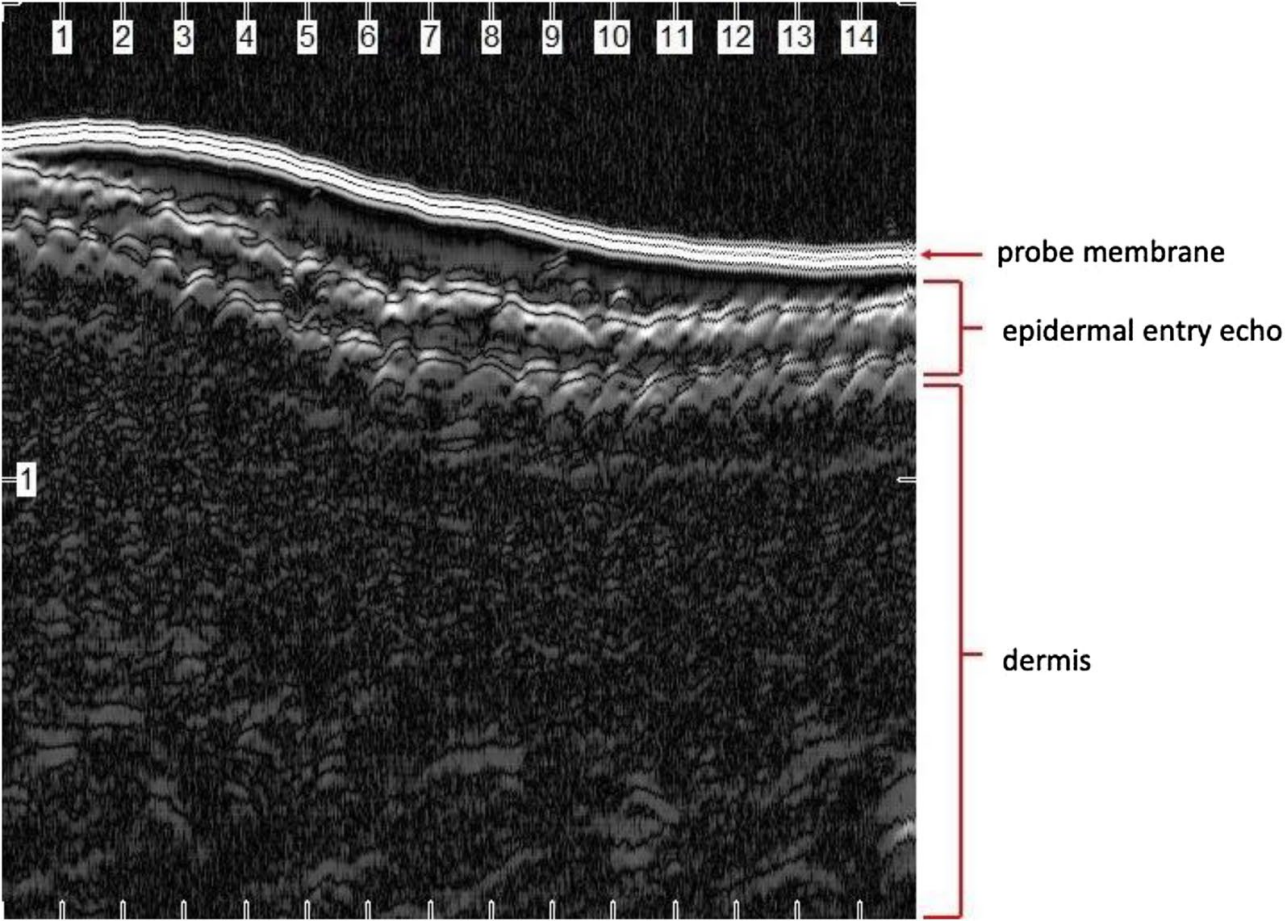
Glabrous skin from the palm using a 50-MHz HFUS probe (Longport, Inc. EPISCAN). Note the bilaminar hyperechoic entry echo due to the thickened stratum corneum in this anatomic location

Compared to non-glabrous skin, glabrous epidermis appears as a bright bilaminar structure when visualized by ultrasound (Fig. 2). The bilaminar structure is thought to be caused by sound waves first entering the thick, compact stratum corneum before entering the normally hyperechoic epidermis itself [9]. This bilaminar appearance can be difficult to visualize at lower frequencies in which the stratum corneum is poorly resolved from the entry echo, but is discernable in UHFUS ranges [31]. Understanding the structure and characteristics of normal glabrous skin has allowed for differentiation between abnormalities in glabrous skin in conditions like Pachyonychia Congenita and other palmoplantar keratodermas [32–34].

At other anatomic sites, hair follicles appear as oblique hypoechoic bands on ultrasound imaging. Multiple studies have demonstrated ultrasound can be used to estimate the phase of hair follicle growth, identify inflammation of hair follicles, and assess hair density [18, 35, 36]. Hair follicle growth phase is estimated based on band termination relative to the subcutis with anagen phase hairs terminating in the deeper dermis and telogen phase hairs terminating more superficially. An inflamed hair follicle will appear thicker and darker relative to a normal hair follicle. A number of studies have suggested that the ultrasonographic findings of an inflamed hair follicle may improve diagnostics by identifying early signs of adnexal pathologies like hidradenitis suppurativa [35, 37, 38].

In addition to identifying changes in hair follicle appearance, ultrasound has also been used to characterize scalp cysts; cysts typically appear as round and dark structures. Trichilemmal cysts characteristically contain hyperechoic areas within their round, dermal anechoic structures, corresponding to the dense keratinous and oily inner-cystic debris [35]. Pilomatricomas appear targetoid with a solid hyperechoic nodule thought to correspond to cystic calcifications or giant cells surrounded by a hypoechoic rim [35].

#### The nail

**Fig. 3:**
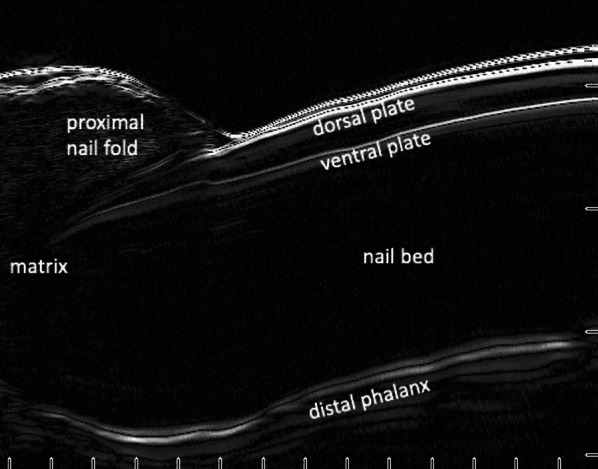
Longitudinal view of a normal nail using a 50-MHz HFUS probe (Longport, Inc. EPISCAN). Note the bilaminar appearance of the nail plate consisting of the hyperechoic dorsal and ventral nail plates separated by a hypoechoic band. The top-most hyperechoic horizontal line represents the probe membrane

The dorsal and ventral plates of the nail are visible as separate entities on ultrasound, generating the second instance of a hyperechoic bilaminar linear structure (Fig. 3). The matrix of the nail is hypoechoic and ventral to the hyperechoic proximal nail fold. The nail bed is normally hypoechoic and just deep to the hyperechoic ventral nail plate. Finally, the surface of the distal phalanx appears as a hyperechoic linear structure [5, 22, 39].

## Ultrasonography in neoplastic disease

### Nonmelanoma skin cancers

**Fig. 4:**
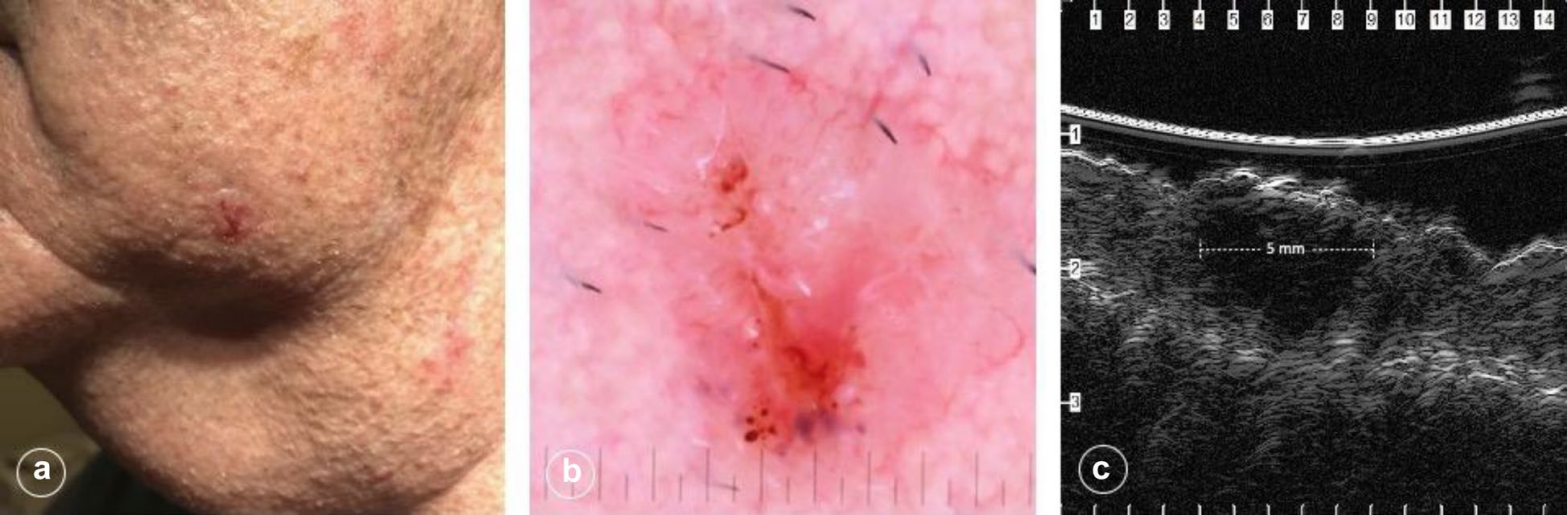
Nodular basal cell carcinoma measuring 5 mm by 7 mm on the left lower cheek of a 66-year-old man. Note the pearly borders and central ulceration in the gross photograph (**a**), the polymorphous vessels and ulceration in the dermatoscopic photo (**b**), and the 5 mm hypoechoic lesion in the superficial dermis across the short axis of this biopsy-supported nodular basal cell carcinoma using a 50-MHz UHFUS probe (Longport, Inc. EPISCAN)

The main dermatologic use of ultrasound is in the pre-operative assessment of non-melanoma and melanoma skin cancers [40, 41] and detection of early neoplasms [42]. In general, cutaneous neoplasms appear sonographically as hypoechoic lesions in a relatively hyperechoic epidermis and/or dermis [43]. The most prevalent human malignancy, BCC, has several histologic subtypes including superficial, nodular, micronodular and morpheaform [44]. The most common BCC histologic subtype—nodular—sonographically appears as a hypoechoic lesion with well-defined borders and is usually found in the dermis, though occasionally can extend deeper into the subcutis (Fig. 4) [42, 45]. Some histologic subtypes of BCC that have demonstrated more aggressive biologic behavior have been shown to feature small hyperechoic dots seen within the hypoechoic tumor. Such spots are referred to as “flower cotton” spots [13, 46]. Though the cause of this artifactual finding is unknown, flower cotton spots are thought to correlate with clusters of apoptotic neoplastic cells within the tumor [13, 46]. Risk of aggressive BCC phenotype, such as micronodular or morpheaform, and risk of recurrence have been correlated to increasing numbers of flower cotton spots [47]. Furthermore, the presence of flower cotton spots is important in differentiating a BCC from melanoma as melanoma lacks these hyperechoic inclusions [48, 49].

The clinical relevance of ultrasound in the examination of suspected SCC is less well defined. Ultrasound has been used to identify SCCs, which also appear as hypoechoic lesions [42, 50]. However, in 2010 Wortsman et al. analyzed 75 non-melanoma skin cancers of which 18 were cutaneous SCCs. She demonstrated a modest but significant increase in diagnostic accuracy with the addition of higher frequencies of ultrasound [9]. An additional study demonstrated the accuracy of SCC measurement with HFUS by comparing SCC depth to histometry, noting a correlation to the 100th of a millimeter [51]. Despite this correlation, ultrasonographic examination of SCCs can be limited by the presence of an acoustic shadow artifact due to the reflection of ultrasound waves by thickened, hyperkeratotic stratum corneum, which may obscure lesion boundaries [48, 52]. Therefore, ultrasound has suggested use as a complementary tool to support the pre-operative evaluation of SCC but not as the sole method of diagnosis [50].

An additional form of non-melanoma skin cancer studied with HFUS is mycosis fungoides (MF) [53, 54]. MF is characterized by presence of a SLEB, which researchers have noticed can be helpful for identifying response to therapy and disease remission; in two separate studies of patients with MF, thinning or complete disappearance of the SLEB corresponded with disease severity and completion of therapy, supporting the utility of HFUS as an objective tool in routine clinical practice [53, 55].

## Melanoma

In the study of melanoma, ultrasound demonstrates a homogenous hypoechoic lesion. The shape of the lesion often correlates to the histologic subtype of the melanoma. For example, nodular melanoma will typically present with a spherical hypoechoic lesion, while a superficial spreading melanoma will present with a thinner, lenticular, hypoechoic lesion subjacent to the epidermal entry echo [13]. Although no distinct ultrasonographic features of melanoma have been described, one important strength of ultrasound is its ability to predict Breslow thickness of a melanoma [48, 56]. This correlation with histologic thickness, the current gold standard on which melanoma staging and surgical planning rests, is predicted to be over 92% [57]. When compared to other techniques like OCT, ultrasound has superior correlation with histology, repeatability and inter-rater reliability [58]. Where OCT tends to underestimate Breslow thickness, ultrasound has been shown to modestly overestimate tumor thickness [58]. The cause of this overestimation is thought to result from the technology’s inability to distinguish an inflammatory infiltrate from melanocytic proliferation and neovascularization. Another contributing factor may be the desiccation of tissue as it is processed for histologic review. Nevertheless, its ability to resolve well beyond the dermis renders HFUS superior to other imaging modalities in predicting Breslow thickness [59].

Though melanomas have been distinguished from benign melanocytic proliferations with specificities as low as 30%, the addition of color Doppler sharply improves ultrasound as a diagnostic tool [43]. Melanomas have been shown to demonstrate more dense vascularization than benign nevi and exhibit low-flow arterioles. In another study of pigmented lesions, the direction of intralesional blood flow demonstrated a 100% specificity in distinguishing melanoma from non-melanoma tumors [46, 60].

## Single-center experience with 50-MHz ultrasound device

In a single-center experience with a 50-MHz device, the Longport, Inc. EPISCAN, the authors examined ultra-high-frequency ultrasound biomicrographs of nearly 70 cutaneous lesions as well as normal skin and adnexae. Transducer power gain and time-gain compensation (TGC) were set to the device’s default settings—100% and 10%, respectively, with a TGC start of 0%. Skin position, or distance between transducer and impermeable membrane barrier, or skin, was 7.5 mm. Scanning depth ranged from 3.8 mm to 15.0 mm, with a scan length of 15 mm at a rate of 1 scan per frame per second. A standard acquisition protocol was followed. The protocol included a horizontal (cranial–caudal) scan and perpendicular scan in the center of each lesion or anatomical area with and without pressure. Then, 3D-views were simulated using two sweeps in both planes over 3–8 s for a total of three to eight 2D-slices in each plane.

Normal skin of the non-sun exposed medial upper arm of a middle-aged man demonstrated a hyperechoic epidermal entry echo and hyperechoic dermis with horizontally arrayed collagen bundles of the reticular dermis. These superficial structures are followed by the hypoechoic subcutis transected by a hyperechoic band of superficial fascia and the hypoechoic biceps muscle invested by the hyperechoic deep fascia (Fig. 1). The thickened stratum corneum over the thenar eminence demonstrated a hyperechoic bilaminar entry echo (Fig. 2). Ultrasound of a first digit nail also demonstrated a bilaminar appearance with hyperechoic dorsal and ventral plates separated by a hypoechoic band (Fig. 3). Finally, a basal cell carcinoma on the medial mandibular cheek of an elderly man demonstrated a hypoechoic lesion in the superficial dermis with subtle posterior acoustic enhancement (Fig. 4).

## Inflammatory skin diseases

### Sclerosing disorders

One of the earliest dermatologic applications of ultrasound was in scleroderma [61]. Due to the pathological increase in collagen deposition, ultrasound can help differentiate between the inflammatory and sclerotic phases of the disease [62]. In the inflammatory phase there is a reduction in echogenicity of the skin and dermal thickening, whereas in the sclerotic phase there is increased echogenicity and thickening [63]. Finally, ultrasonographic features of sclerodermatous skin in the atrophic phase demonstrates thinning of the dermis with an echogenicity similar to unaffected surrounding skin [64].

The modified Rodnan score is used to measure skin hardening. Skin lesions are scored on a 4-point scale from 0 to 3. This score is applied to 17 discrete areas of the body after evaluation by a clinician. A study found that 18-MHz ultrasound is more sensitive than clinical assessment in measuring skin hardening, with results of ultrasound providing the additional benefits of being quantitative, valid, and reproducible [65].

In a study of 16 patients with morphea and four with extragenital lichen sclerosis, the results of HFUS demonstrated unique features of extragenital lichen sclerosis characterized by widened, polycyclic, hyperechoic and disorganized epidermal entry echo relative to that seen in morphea [62]. Another study using UHFUS to measure response to treatment demonstrated a statistically significant decrease in dermal thickness and density after treatment with ultraviolet A1 phototherapy [66].

### Psoriasis

Currently, evaluation of psoriasis in a clinical setting includes the use of the Psoriasis Epidemiology Screening Tool, a validated screening tool designed to identify patients who should be referred to a rheumatologist for possible co-existing psoriatic arthritis. Rheumatologists, on the other hand, use the Classification Criteria for Psoriatic Arthritis (CASPAR) to characterize psoriatic arthritis disease burden.

A 2015 study used ultrasound (6–18 MHz) to determine the prevalence of enthesitis, a feature in the pathogenesis of psoriatic arthritis, among psoriasis patients with and without psoriatic arthritis as defined by CASPAR criteria [67]. The study found at least one ultrasonographic sign of enthesitis in approximately half the patients with psoriatic arthritis, but also in almost half of the psoriasis patients without a diagnosis of psoriatic arthritis [67]. Furthermore, after these patients were treated for 6 months with systemic therapy, ultrasonographic features of enthesitis in both of these groups improved, suggesting subclinical joint involvement and underdiagnosis of psoriatic arthritis among psoriasis patients [67].

In a 2013 study, researchers found that 78% of patients with psoriasis had at least one nail abnormality identified by ultrasound: loss of ultrasonographic architecture, pitting, or both [68]. This study suggested that OCT was better than ultrasound at 9–14 MHz for identifying features of psoriatic nails, but both were more sensitive than clinical examination alone [68]. In a later study utilizing a UHFUS 40-MHz probe, researchers identified ventral nail plate deposits, irregular or fused nail plates, and significantly thicker nail plates in patients with psoriasis compared to controls, with Doppler-ultrasound noting increased nail blood flow and increased vascular resistance [69].

Finally, a recent study using 20-MHz HFUS to compare two treatment arms reliably demonstrated a reduction in the SLEB of psoriatic skin coinciding with clinical improvement, supporting the use of ultrasonography as a useful objective marker in patients with psoriasis [70].

### Hidradenitis suppurativa

Hidradenitis suppurativa (HS) is an inflammatory disease that affects between 0.05 and 4.1% of the population [71–73]. This disease results in chronic inflammation of the hair follicle and presents with painful, erythematous nodules that often rupture, forming draining sinus tracts that eventually scar. Current HS staging tools include Hurley staging, HS severity index, HS physician global assessment and sonographic scoring of HS [74, 75]. Hurley staging is generally the most commonly used method for assessment of HS severity, but lacks the ability to effectively measure disease fluctuation and response to treatment [74].

A number of studies have demonstrated that these various staging tools underestimate the true disease severity in comparison to ultrasound-assisted staging of HS [76–79]. Ultrasound has allowed for understanding of a proposed sequence of skin changes in HS pathology with the earliest sign of follicular widening eventuating in the formation of fistulous tracts and increased vascularity over acutely inflamed lesions [78]. A meaningful association between the ultrasonographically measured diameter of an HS nodule and the patient’s subjective assessment of the nodule’s tenderness and the clinician’s assessment of erythema have also been established [80].

Finally, ultrasound can aid in medical and surgical management of HS. One study noted a modification in the management of 82% of cases following ultrasonographic evaluation with a 24% transition from medical to surgical management [78].

### Atopic dermatitis

Atopic dermatitis (AD) is a chronic inflammatory skin condition characterized by intense pruritis and abnormal skin barrier function [81]. Currently, there is a lack of standardization of core outcome measurement tools for this disease. The Harmonising Outcome Measures in Eczema initiative recommends the use of the Eczema Area and Severity Index and the Scoring Atopic Dermatitis in assessing physician-reported signs of AD, noting the utility of the latter in clinical trials [82, 83]. But these disease severity tools are subjective, cumbersome and have demonstrated poor reproducibility [84, 85].

A number of studies have attempted to better understand the severity of AD through the use of ultrasound. In particular, these studies have sought to measure the thickness of the SLEB and skin echogenicity in order to quantify disease burden. Unlike in sun damaged skin, in AD, the SLEB appears to be the result of edema and inflammatory cell infiltrate in sites of active inflammation [13]. In one 2019 study, the mean thickness of the hypoechoic band was found to be higher in lesional skin but present in both lesional and non-lesional skin of individuals with AD [81]. A separate study compared SLEB thickness in patients with AD as visualized by 20-MHz ultrasound to their EASI score and histopathological findings from 5 mm punch biopsies. The study found SLEB thickness correlated with the histologic degree of epidermal hyperkeratosis, parakeratosis, spongiosis, and intensity of inflammatory infiltrates as well as the provider-assessed EASI scores [84]. Finally, two studies using HFUS and UHFUS to examine the effect of phototherapy and tacrolimus on SLEB thickness found statistically significant reductions in mean SLEB thickness [86, 87].

### Granulomatous diseases

Sarcoidosis is characterized by the formation of granulomas in a number of different organs and is associated with cutaneous induration and erythema. A pilot study in 2017 evaluated the utility of UHFUS in objectively quantifying granuloma burden in cutaneous sarcoidosis in comparison to the Cutaneous Sarcoidosis Activity and Morphology Instrument (CSAMI) and histopathological evaluation [88]. The study found a strong correlation between the mean brightness of UHFUS and the lesion CSAMI score, as well as the percent of the dermis with granulomas on histopathology; ultimately, these results highlighted the utility of ultrasound in quantifying disease burden in patients with cutaneous sarcoidosis [88].

Another granulomatous disease, idiopathic facial aseptic granuloma (IFAG), is a self-limiting pediatric disease; as such, it does not require aggressive treatment [16]. On ultrasound, IFAG can appear as a poorly defined oval shaped hypoechogenic lesion in the dermis surrounded by areas of hyperechogenicity and hypervascularization due to inflammation. Ultrasound can therefore be utilized to differentiate IFAG from its differential diagnoses that might require further management. For example, a facial abscess may exhibit a characteristic “squish sign” with ultrasound and would require incision and drainage with antimicrobial therapy [89]. Further differentiating factors include lack of calcium deposits, differentiating it from a pilomatricoma, lack of defined borders, as seen in epidermal cysts, and lack of vascularity, as seen in vascular tumors [16]. The use of ultrasound to properly diagnose, manage and monitor disease progression in IFAG is therefore important and provides a non-invasive alternative to biopsy [16].

## Vascular lesions

A number of vascular lesions have been studied using ultrasound, including Port-wine stains (PWS), arteriovenous (AV) malformations and spider nevi. The first study to successfully examine PWS using ultrasound was conducted in 2018 [90]. This retrospective study utilized both 10-MHz and 22-MHz transducers with and without Doppler to assess lesional depth, echogenicity, vessel density and AV flow signals. The results of this study demonstrated a clear contrast between lesional and normal skin [90]. Furthermore, the study demonstrated ultrasound could help differentiate a PWS from other vascular anomalies due to their hypoechoic, poorly vascularized appearance with venous flow, while other capillary-arteriovenous malformations appear hyperechoic and well-vascularized with mixed arterial and venous blood flow [90].

Another type of vascular lesion known as a Spider nevus has also been characterized using ultrasound [91]. Spider nevi were historically classified as low-flow capillary malformations [92]; however, a 2017 study by Alegre-Sanchez et al. found that spider nevi demonstrated pulsatile flow on dermoscopy and ultrasound. This finding was subsequently supported by the presence of high-flow arterial-type waveform as seen on Doppler-ultrasound, suggesting that spider nevi are more similar to arteriovenous malformations, or high-flow vascular lesions [91].

Ultrasound can also be used to guide and assess response to treatment [48]. For example, researchers have used its Doppler capabilities to correctly identify venous structures and aid in guiding injectable foam sclerotherapy for the treatment of venous leg ulcers, demonstrating good healing rate and a low reflux ratio [93].

## Cosmetic applications

Several cosmetic applications of ultrasound have been established. For example, botulinum toxin injections demonstrate increase subcutaneous echogenicity leading to a blurring of the normally discrete boundaries between the subcutis and underlying muscle [94]. Similarly, fillers can be visualized using ultrasound to determine locations of prior injections—an important application since a small but significant proportion of patients seeking injectable fillers fail to report prior procedures. The risk of granulomas and filler dermopathies associated with injections of multiple filler materials has been established [95–97].

The efficacy of topical vitamin C therapy in increasing collagen production, reducing ultraviolet radiation induced oxidative stress and enhancing dermal remodeling has been demonstrated in several studies [98, 99]. One notable study using HFUS evaluated the skin of patients throughout a 60-day period of topical vitamin C therapy, demonstrating increased echogenicity in both the epidermis and dermis after 40 days and to a greater extent after 60 days [98]. Ultrasound at 15-MHz and 20-MHz has also been used to evaluate the efficacy of an oral micronutrient supplement on preservation of skin quality through the measurement of skin thickness and density [99, 100].

## Conclusion

While ultrasound has been used in dermatology for more than 40 years, its uses have continued to evolve with improvements in technology. Today, there are a few dermatologic lesions with pathognomonic sonographic features. One example is the “jellyfish” sign described in some cases of dermatofibrosarcoma protuberans [101]. Other examples include the “saw-tooth” pattern of cellulite [102–104]. Pathognomonic ultrasonographic features are likely to increase with integration of higher frequency ultrasound devices in clinical practice; for future work with HFUS, a core international task force group formed by 15 physicians from 11 countries called the DERMUS (dermatologic ultrasound) group recommends dermatologists use Doppler-capable ultrasound devices with a frequency of at least 15- to 22-MHz in all ultrasound examinations of the skin [18].

While there is support for use of ultrasound in future dermatological practice, there are a number of limitations associated with the use of this tool. One major limitation is the skill of the operator. Consequently, the DERMUS group suggests utilizing ultrasound at least 300 times per year to achieve minimum competency. Similarly, it is strongly advised that the dermatologist functions as the sonographer because of the years of training in dermatologic pathologies and ability to correlate histology with clinical presentation of cutaneous lesions [18].

As frequency advancements in ultrasonography continue, the broad applications of this imaging modality will continue to grow. Ultrasound is a fast, safe and readily available tool that can aid in diagnosing, monitoring and treating dermatologic conditions by providing more objective assessment measures.

## Data Availability

Not applicable.

## Abbreviations

HFUS: High-frequency ultrasound
MHz: Megahertz
TBDP: Total body digital photography
RCM: Reflectance confocal microscopy
OCT: Optical coherence tomography
μm: Micrometers
BCC: Basal cell carcinoma
SCC: Squamous cell carcinoma
SLEB: Subepidermal low-echogenic band
MF: Mycosis fungoides
CASPAR: Classification criteria for psoriatic arthritis
HS: Hidradenitis suppurativa
AD: Atopic dermatitis
CSAMI: Cutaneous Sarcoidosis Activity and Morphology Instrument
IFAG: Idiopathic facial aseptic granuloma
PWS: Port-wine stain
AV: Arteriovenous
